# Introduction of soft drinks and processed juice in the diet of infants attending public day care centers

**DOI:** 10.1016/j.rpped.2014.06.009

**Published:** 2015-03

**Authors:** Giovana Longo-Silva, Maysa Helena de Aguiar Toloni, Risia Cristina Egito de Menezes, Leiko Asakura, Maria Alice Araújo Oliveira, José Augusto de Aguiar Carrazedo Taddei

**Affiliations:** a Universidade Federal de Alagoas, Maceió, AL, Brazil; b Universidade Federal de Lavras, Lavras, MG, Brazil; c Universidade Federal de São Paulo, São Paulo, SP, Brazil

**Keywords:** Industrialized foods, Food habits, Food consumption, Child day care centers, Infant

## Abstract

**OBJECTIVE:**

Identifying at what age infants enrolled in public day care centers are introduced to soft drinks and industrialized juice, as well as comparing the nutritional composition of these goods with natural fruit juice.

**METHODS:**

A cross-sectional study with the mothers of 636 children (aged 0 to 36 months) from nurseries of day care centers, who were asked questions about the age of feeding introduction. This study evaluated the proximate composition of soft drinks and artificial juice, comparing them with those of natural fruit juice regarding energy, sugar, fiber, vitamin C, and sodium values. The chemical composition of fruit juice was obtained by consulting the Table of Food Composition and, for industrialized drinks, the average nutritional information on the labels of the five most consumed product brands.

**RESULTS:**

The artificial drinks were consumed before the first year of life by more than half of the children studied, however, approximately 10% consumed them before the age of 6 months. With regard to the comparison among the drinks, artificial fruit juice beverages and soft drinks proved to contain from nine to 13 times higher amounts of sodium, and 15 times less vitamin C than natural juices.

**CONCLUSIONS:**

The introduction of soft drinks and industrialized juice in the diet of infants was inopportune and premature.. When compared to natural fruit juice, these have inferior nutritional composition, which suggests the urgent need for measures based on strategies for food and nutrition education in order to promote awareness and the maintenance of healthy eating habits.

## Introduction

Healthy eating habits started in childhood not only bring immediate health benefits, but also influence future practice and preferences, and are associated with health protection in adulthood.^1^ In this context, exclusive breastfeeding during the first six months of life is recommended as a public health measure, and, after that period, the introduction of complementary foods, with continuation of breastfeeding until 2 years of age or older, as well as discouraging the offer of processed foods in the first years of life.^2^

In spite of the indisputable benefits of the practical application of such recommendations, several studies have shown that contemporary society tends towards inadequate dietary patterns, with an impact on the early introduction of processed and ultraprocessed foods in childhood diet.^3^^-^ 6 This fact is a direct consequence of women entering the labor market, together with the lack of time for food preparation and the confidence given to products advertised by the media, and associated by the latter specifically to children.^7^

Especially regarding liquid foods, there has been an increase in the consumption of artificial beverages such as soft drinks and processed juices. Moodie et al^7^ assessed trends in the acquisition of soft drinks in low- and middle-income countries, including Brazil, and high-income countries, showing an emphatic annual growth of *per capita* volume consumed between the years 1997 and 2009, with an increase of 5.2% in low- and middle-income countries and 2.4% in high-income countries, demonstrating that it is a global problem that does not depend on the socioeconomic and cultural setting.

It is noteworthy that, in addition to the immediate damages caused by the consumption of such beverages, such as impaired intake of breast milk and other healthy foods and the nutritional adequacy of micronutrients, their presence in the habitual diet can have an impact, in the medium- and long-term, on the increase in overweight, obesity, and associated chronic diseases,^8^ as verified by Boynton et al^9^ in 548 children in Massachusetts, whose body mass index (BMI) and prevalence of obesity increased for each additional serving of beverages containing added sugar.

Given the above, the present study aimed to identify the age of introduction of soft drinks and industrialized juices in the diet of infants enrolled in public daycare centers and nurseries, and to compare the nutritional compositions of these drinks with natural fruit juice.

## Method

This was a multiple observational study, with the first observation in the second semester of 2007 and the second observation in the second semester of 2010. The study was developed in the nurseries of eight public daycare centers belonging to the Education Coordination of Santo Amaro district in the city of São Paulo, which were part of the "Projeto Crecheficiente" - "Impact of teacher training in public/philanthropic daycare centers on hygienic-dietary practices and health/nutrition of infants", which aimed to train, develop, and refresh the knowledge of daycare professionals regarding health care and nutrition provided to infants and to evaluate the acquisition of knowledge by teachers related to the activities performed by them. The selection process of the daycare centers and the adopted criteria are described in another publication.^10^

Of the eight daycare centers selected for the study in 2007, one was excluded in 2010 due to lack of interest in joining the study during the data collection period. The study consisted of all enrolled children, totaling 636, with 270 in 2007 and 366 in 2010, of both genders, aged between 4 and 38 months, who regularly attended the nurseries of selected daycare centers and who received authorization from parents or guardians to participate in the study by signing the informed consent. With the purpose of standardizing the data analysis, it was decided to jointly analyze data from the cross-sectional observations of 2007 and 2010, as it was not the objective of this study to compare the collected data, but rather to create a large sample including all infants that attended the nurseries of the assessed daycare centers.

The introduction of two types of food, soft drinks and processed/artificial juice, was assessed from data collected using a structured, pre-coded, and pre-tested questionnaire regarding their content and construct validity. The age in months at which soft drinks and processed juice were introduced was recorded and juice categories were not investigated. Data collection was carried out in 2007 and 2010 by four properly trained nutritionists, who interviewed the parents or guardians of the children in the daycare nurseries on scheduled days. 

Aiming to standardize the completion of the tool, the authors prepared a manual that included guidelines to interviewers and coding of variables. Data regarding their internal consistency was analyzed, followed by double entry and validation. Statistical analyses were performed using the Epi Info 7-2012 program (Centers for Disease Control and Prevention - Georgia, United States). To determine the age of introduction of soft drinks and processed/artificial juice, the authors used the cumulative percentage frequency in age groups 0-6, 7-12, and 12-36 months.

The proximate composition of the soft drinks and processed juice was evaluated, comparing them the natural juice for energy content, total carbohydrate, fiber, vitamin C, and sodium content. Orange-flavored soft drinks and juice were selected, as they are the most often consumed in Brazil.^11^

The proximate composition of the natural juice was obtained by consulting the Brazilian Table of Food Composition.^12^ To define the proximate composition of the processed foods, the authors used the means of nutritional information found on the labels of the five most consumed brands of the two products,^13^ whereas for the category of processed/artificial juice, the following types were considered: artificial powdered drink mixes, fruit nectars, and sweetened processed juice.^14^

This study was approved by the Research Ethics Committee of Universidade Federal de São Paulo (UNIFESP) (CEP 0471/10).

## Results

The median age of the children was 23 months. Of all the children studied (n=636), there was a predominance of males (55.7%) and a higher proportion of mothers aged 20-35 years, with a mean age of 28±6.5 years. Regarding maternal education, it was observed that 36% of the mothers had less than eight years of study (Table 1). 

**Table 1:** Demographic and socioeconomic characteristics of the studied children.

	n	%	Median
Child’s age			
0 to 6 months	4	0.6	23 months
7 to 12 months	50	7.9	
13 to 36 months	577	91.3	
≥36 months	1	0.2	
Gender			
Male	354	55.7	—
Mother’s age			
<20 years	51	8	28 years
Maternal schooling			
<8 years	171	26.9	10 years
Family income (MWs)			
<1	21	3.3	
1.0-2.0	265	41.9	1.9 MWs
2.0-3.0	137	21.6	
≥3.0	210	33.2	

MWs, Minimum wages in reais during the study period, equivalent to R$380 in 2007 and R$510 in 2010.

Table 2 shows the cumulative frequency (%) by age of introduction of soft drinks and processed/artificial juice, as well as the mean and standard deviation of age at introduction. It can be observed that, for more than half of the assessed children, soft drinks and processed juice were offered by the end of the first year of life, and less than 10% of the children had not consumed these beverages by 36 months of age. 

**Table 2:** Cumulative percentages by age range, mean, and standard deviation of the introduction of soft drinks and processed/artificial juices.

		Soft drinks	Processed/artificial juices^b^
Months at introduction			
0-6	%	7.4	14.3
7-12	%^a^	53.8	62.9
12-36	%^a^	90.4	91.8
Did not introduce	%	9.3	7.9
Did not introduce	%	0.3	0.3
Mean±SD	Months	15.9±7.8	14.0±7.9

^a^Accumulated percentage. ^b^Related to the five most often consumed brands of artificial juice powder and sweetened processed fruit juices.

**Figure 1:**
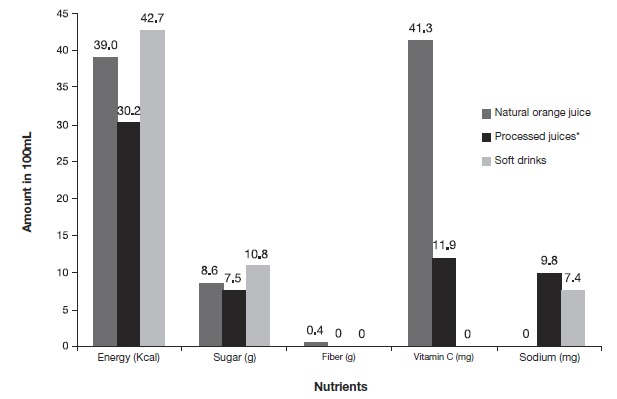
Comparison of energetic value, sugar, fiber, vitamin C, and sodium content in the chemical composition of natural fruit juice, processed/artificial juice, and soft drinks.^12-14^ *Regarding the five most often consumed brands of artificial juice powder and sweetened processed fruit juices

Table 3 shows the nutritional composition of orange juice and the mean and standard deviation of soft drinks and processed juice. The comparison of the nutritional compositions are shown in Figure 1, where it demonstrates that artificial juices and soft drinks contain 9-13 times higher amounts of sodium and 15 times less vitamin C, compared to natural juice. Additionally, both artificial beverages lack fiber content.

**Table 3:** Energetic value, sugar, fiber, vitamin C, and sodium content in the centesimal composition of natural orange juice, processed juices, and soft drinks.^12-14^

	Natural juice^a^	Processed juices^b^ Mean±SD	Soft drinks Mean±SD
Energetic value (Kcal)	39	30.2±18.4	42.7±1.8
Sugar (g)	8.6	7.5±5.3	10.8±0.6
Fiber (g)	0.4	0	0
Vitamin C (mg)	41.3	11.9±10.2	0
Sodium (mg)	Trace^c^	9.8±6.1	7.4±1.9

^a^ Natural orange juice. ^b^ Regarding the five most often consumed brands of artificial juice powder mixes and sweetened processed fruit juices. ^c^ Trace, trace amounts, values below measureable limits.

## Discussion

It should be emphasized that the decision to compare processed/artificial juice and soft drinks with natural juice is because the latter is part of the list of the liquid foods recommended in complementary feeding, although it is emphasized that according to the recommendation of the Ministry of Health for the practice of breastfeeding, natural juice should not be offered before six months of age.^2^

The results showed an early and untimely introduction of soft drinks and artificial juices in the diet of the assessed children, and more than half consumed them before the first year of life. It was also verified that 7.4% and 14.3% of the mothers, respectively, offered soft drinks and processed juice before the sixth month of life; these are considerable percentages, as exclusive breastfeeding is recommended in this period.

Given that the early introduction of complementary foods, regardless of their composition, is already mentioned in the literature as a risk factor for the reduction in the duration and frequency of breastfeeding,^2^ interaction with nutrient absorption, risk for diarrheal and respiratory diseases, child mortality, and impact on growth,^15^ when the introduction includes obesogenic foods, there are even more severe health risks, especially in regard to the predisposition to obesity and chronic noncommunicable diseases (NCDs), including diabetes, arterial hypertension, and cardiovascular diseases. 

The abovementioned data reflect trends in eating behavior in the contemporary society. It is verified that, with the liberalization of the economy and monetary stabilization, the consumer market has expanded.^16^ Simultaneously with the increase in purchasing power, the real price of processed foods has decreased, encouraging greater participation of the lower income strata. In general, as the *per capita *income of a country increases, the degree of sophistication in food consumption also increases and the population chooses more elaborate foods, such as processed foods.^16^^,^ 17

Corroborating this assertion, data from the 2008-2009 Family Budget Survey^18^ showed that consumption of soft drinks and processed juices/powdered drink mixes/artificial juices had the highest mean *per capita* daily consumption, with 94.7 and 145mL/day, respectively, with no differences between income groups, and were associated with a lower intake of vitamin C and fiber, and higher energy and sodium content. However, the recommended amount of fruits and vegetables/day is not achieved even at the 90^th^ percentile of the Brazilian population, reflecting the preference for industrialized food. Specifically regarding the child population, Spinelli et al^5^ found the presence of food considered to be unnecessary in the diet of 400 children younger than one year treated in Basic Health Units in a municipality of the Greater São Paulo, and verified that 58.9% of the children consumed soft drinks, and 14.5% started consuming them between 4 and 6 months of age.

Similarly, in a prospective study, Caetano et al^4^ identified through a seven-day food record applied to parents, in samples of infants living in Curitiba, São Paulo, and Recife, that the weekly frequency of soft drink intake was 9% and 20.7% of processed/artificial juice. These findings, in addition to the presence of other foods inappropriate for the age range, reflect a quantitative micronutrient inadequacy higher than 15%, 40%, and 10% for calcium, iron, and vitamin C, respectively.

Moreover, Silva et al^6^ analyzed data from Brazilian population-based surveys and found that, among 3,789 children younger than five years, 70% had consumed soft drinks and processed/artificial juice at least once during the seven days prior to the interview, with a reported daily consumption by 22% of the assessed children. The authors emphasized that the prevalence was higher among residents of urban areas, possibly justified by the recent marketing of a large variety of such drinks, easy access to stores, as well as the appeal of advertising campaigns. 

Analyzing the data from this study, with focus on the early age of introduction, these data become even more of a concern, when one considers the trend of increased consumption of soft drinks and artificial juices with age,^19^ suggesting that these children may remain exposed to these products habitually and increasingly as they grow. 

As an example, Dubois et al^20^ followed a cohort of more than two thousand children, aged 2 to 5 years, and found that the proportion of those who consumed soft drinks once a week between meals increased with age (42% at 2.5 years, 47% at 3.5 years, 48% at 4.5 years), discussing the possible association of this trend with greater autonomy of the children, as they start school and are exposed to the school cafeterias in public and private schools, as well as the possible belief of the parents regarding the minimized harmful effects in older children. In agreement with that, Lopes et al^21^ also observed a direct association between nutritional status and soft drink consumption among children in São Paulo, in which 83.2% of those that consumed soft drinks every weekend were overweight and 76.6% were obese. 

The abovementioned data can be explained by the inadequate nutritional composition of this type of beverage, noting that the amount of carbohydrates present in 100mL, corresponding to 10.8 g, consists exclusively of added sugar, which corresponds to more than 80% of the daily recommendation proposed by WHO for children aged 1 to 3 years (13.3g/day).^22^ Although the amount of sugar added to artificial juice is unknown, as the presence of this information on the label is not mandatory,^23^ it is assumed that it is also present in excessive amounts.

The definition of the term added sugar includes, in addition to the mono- and disaccharides, some oligosaccharides, and does not consider some sugars naturally present in foods, such as fruits. These sugars are those added to prepared and processed foods with the aim of increasing palatability and providing better viscosity, texture, color, and durability.

This term includes refined white sugar, brown sugar, high-fructose syrup, glucose syrup, liquid fructose, fructose-based sweetener, honey, and molasses.^24^ Its intake is associated with reduced overall diet quality, early occurrence of overweight and obesity, development of chronic diseases and their risk factors,^2^ as well as contribution to the development of dental caries, a fact verified by Biral et al,^25^ who evaluated the children participating in the 2007 data collection of this study and identified 77% of them with some alteration according to the modified caries index (ceo-mod ≥1) and 72.37% with plaque.

Supported by the deleterious effect of high intake of sugar and artificial drinks, and with the specific focus of prevention and control of overweight and dental caries, in 2014 the WHO proposed reducing the maximum recommended limit of sugar, from 10% to 5% of total daily energy intake.^21^ Contrary to these perceptions, the Brazilian population, the world's largest consumer of sugar, has an estimated intake equivalent to 16.4% of total calories.^26^ According to Levy et al,^26^ the participation of white sugar in the past 15 years has been reduced, while the contribution of sugar added to foods has doubled, primarily through the consumption of soft drinks and cookies.

Although not widely known to global consumers, the difference between real juice, nectar, and refreshment is related to the content of fruit juice in the bottled beverage. In the nectar category, the bottled drink has a lower content of pure juice and may contain sweeteners, food colorings, and preservatives, as well as additives,^14^ which are generally cheaper than the soluble solids of the fruit, making it more accessible to an intermediate *per capita* income consumption category.^27^ The normative order of the Ministry of Agriculture, Livestock, and Supply (Ministério da Agricultura, Agropecuária e Abastecimento [MAPA]) published in the Official Gazette, in 2012, increased from 30% to 50% the minimum content of orange juice in beverages sold as fruit nectar.^28^

In the category of refreshment, the content in the bottled juice is 30% volume of natural juice. These drinks have a greater amount of additives, making them a product of lower added value, representing the gateway to the consumption of processed fruit drinks by the low-income population.^27^ Orange sodas should contain, obligatorily, a minimum of 10% in volume of the respective juice in its natural concentration, and solid preparations for artificial juice do not have raw material of plant origin in their composition.^14^ The lack of these definitions and the lack of clarity of the labels of these foods can cause confusion to the consumer of such beverages, who is under the illusory prospect of adequately replacing natural juice or even fresh fruit.

Regarding the quantities of micronutrients observed for soft drinks and artificial juices analyzed, the amount of vitamin C was approximately four times higher in the chemical composition of the natural juice (41.3mg) when compared to artificial juice (11.9mg), and zero vitamin C was found in the soft drink, emphasizing that this vitamin plays an important role in the absorption of nonheme iron and for the immune system. Both drinks do not offer any amount of dietary fiber.

Additionally, the potential inhibitory role of these drinks in the absorption of other micronutrients is emphasized. Soft drinks contain polyphenols in their composition, which also play an inhibitory role in the absorption of nonheme iron^29^ contributing to the risk of developing iron-deficiency anemia, a nutritional deficiency of worldwide public health concern, especially at the age of the children in the present study. Specifically in the present population, Konstantyner et al^10^ found a prevalence of anemia of 51.9% (95% CI: 44.9 to 58.8%) in 2007.

Calcium is another nutrient that may show deficiency as a result of the intake of these beverages. There are several reasons for the hypothesis that carbonated soft drinks, particularly cola drinks, may be associated with lower bone mineral density (BMD), highlighting the presence of caffeine, identified as a risk factor for osteoporosis.^30^ The phosphoric acid interferes with the micronutrient absorption and contributes to imbalances that increase their excretion; moreover, corn syrup, which has high-fructose content and is used to sweeten these beverages, has a negative effect on bones.^30^

Demonstrating these assumptions, Tycker et al,^31^ using data from the Framingham Osteoporosis Study, assessed the association between consumption of carbonated beverages and bone mineral density, with a subdivision between cola and non-cola types. These data were obtained by dual energy X-ray absorptiometry and application of the Dietary Intake Frequency Questionnaire. The mean bone mineral density (BMD) in women with a daily intake of cola was 3.7% lower at the femoral neck and 5.4% at Ward's triangle, when compared to those who consumed <1 serving of cola/month, with statistical significance (*p*<0.001-0.05).

Also demonstrating the unfavorable aspects of early consumption of these beverages, although not among the objectives of this study, it should be emphasized that these artificial drinks have food additives in their composition which, according to recommendations by the WHO and the United Nations Food and Agriculture Organization (FAO), should not be used internationally in products intended for children under 1 year.^32^ Nogueira^8^ evaluated the use of food colorings by preschool children in public and private daycare centers in Rio de Janeiro and found an excessive contribution from soft drinks, bottled juices, and refreshments, whose intake was higher among children in public daycare centers, due to the low cost of these products.

In addition to the dangers associated with the nutritional aspect of artificial juices and soft drinks, it should also be emphasized that these children are starting to consolidate their eating habits. Although the desire for the sweet taste is innate, its consolidation is also influenced by experience, i.e. by repeated consumption of sweet foods during infancy.^33^

Therefore, considering all the above adverse effects, it is important to understand the factors that contribute to the early provision of artificial beverages in the home environment. In addition to representing a reflection of the widespread poor dietary habits of the population, there have been striking changes in the food acquisition profile of the families and their consumption of pre-prepared meals, further reinforcing the influence of advertising and their trust in the products presented by the media and advertisements, mainly due to misinformation about the risks to health and nutrition associated with early and continued consumption of these foods.^7^

Regarding the role of food advertising that encourages the consumption of these products, although the American Dietetic Association recommend that children younger than 2 years should not exposed to television or other screen or electronic devices, on average, they watch televised programs for one to two hours each day, and 14% of children between 6 and 23 months watch two or more hours a day,^34^ reflecting that these children may continue under the same context of vulnerability to food advertising.

Considering this scenario, the "Strategic Action Plan to Tackle Non-Communicable Chronic Diseases (NCDs) (2011-2022)" was published in 2012, establishing as its main actions the promotion of healthy eating. Among the goals specified is the implementation of agreements with the food industry to reduce the salt and sugar contents of processed foods, plus restrictions on the marketing of foods and beverages high in salt, fat, and sugar, especially to children.^35^

While the agreement between the public and private sectors seems a promising strategy, Moodie et al^7^ reported that the food industry has been employing a series of artifices contrary to public health programs and policies, such as funding research and health conferences, sponsoring events related to physical activity, and creating opposition to regulations using the argument that government intervention is coercive and oppresses freedom of choice and individual responsibility. Under this approach, and therefore, with less support from the civil society, the adoption of regulatory measures, such as restrictions, warnings, and increased taxes and fees on industrialized products becomes more cautious and even unfeasible. 

Regarding the limitations of the present study and considering its cross-sectional design, data related to the introduction originate from information provided by the mothers or guardians through recall, which could imply inaccuracies or biases. However, it is believed that the results were not significantly influenced, as the introduction occurred only a few months before the interviews. Another limitation includes the use of data from food composition tables and nutritional labels of food products, noting that the National Health Surveillance Agency (ANVISA) allows a variation in the nutrient content between the nutritional labels and actual values, of up to approximately 20%.^22^

Finally, it can be concluded that the introduction of soft drinks and processed juices in the diet of infants is untimely and premature, and when compared to natural fruit juice, they show inferior nutritional composition. These findings suggest the need for actions, based on food and nutrition education strategies directed at parents, children, and day care employees, which can help to reduce the consumption of artificial beverages, aiming to promote the formation and maintenance of healthy eating habits, positively contributing to the adequate growth and development of children and preventing short-, medium-, and long-term increases of overweight, obesity, and chronic diseases.
