# Supplementary material for: Introduction of soft drinks and processed juice in the diet of infants attending public day care centers
Source: Rev Paul Pediatr. 2015 Mar;33(1):34–41. doi: 10.1016/j.rpped.2014.06.009 (PMC4436954; doi:10.1016/j.rpped.2014.06.009)
Supplement: Translation

# Translation: Introdução de refrigerantes e sucos industrializados na dieta de lactentes que frequentam creches públicas

**Authors:** Giovana Longo-Silva, Maysa Helena de Aguiar Toloni, Risia Cristina Egito de Menezes, Leiko Asakura, Maria Alice Araújo Oliveira, José Augusto de Aguiar Carrazedo Taddei

**Affiliations:** a Universidade Federal de Alagoas (UFAL), Maceió, AL, Brasil; b Universidade Federal de Lavras (UFLA), Lavras, MG, Brasil; c Universidade Federal de São Paulo (UNIFESP), São Paulo, SP, Brasil

**Keywords:** Alimentos industrializados, Hábitos alimentares, Consumo de alimentos, Creches, Lactente

## Abstract

**OBJETIVO:**

Identificar a idade de introdução do refrigerante e de sucos industrializados na dieta de lactentes matriculados em berçários de creches públicas e comparar as composições nutricionais dessas bebidas com as do suco de fruta natural.

**MÉTODOS:**

Estudo transversal com 636 crianças (de zero a 36 meses) de berçários de creches, cujas mães foram entrevistadas sobre idade de introdução dos alimentos. Avaliaram-se as composições centesimais do refrigerante e sucos industrializados, comparando-as com as do suco de laranja natural para valor energético, açúcar, fibra, vitamina C e sódio. A composição centesimal do suco de laranja foi obtida por meio de consulta à Tabela de Composição de Alimentos e, para as bebidas industrializadas, utilizaram-se as médias das informações nutricionais contidas nos rótulos de cinco marcas mais consumidas dos produtos.

**RESULTADOS:**

O refrigerante e suco industrializado foram consumidos antes do primeiro ano de vida por mais da metade das crianças estudadas, sendo que cerca de 10% o consumiram antes dos seis meses. Quando comparadas à composição do suco de laranja natural, bebidas forneceram quantidades de 9 a 13 vezes superiores de sódio e 15 vezes inferiores de vitamina C.

**CONCLUSÕES:**

A introdução de refrigerantes e sucos industrializados na dieta dos lactentes foi inoportuna e precoce. Comparados ao suco de fruta natural, tais bebidas possuem composição nutricional inferior, sugerindo a necessidade de medidas fundamentadas em estratégias de educação alimentar e nutricional como forma de promover a formação e manutenção de hábitos alimentares saudáveis.

## Introdução

Padrões alimentares saudáveis iniciados na infância não só trazem benefícios imediatos à saúde, mas também influenciam as práticas e preferências futuras e associam-se à proteção da saúde na vida adulta.^1^ Neste contexto, recomenda-se, como medida de saúde pública, o aleitamento materno exclusivo durante os primeiros seis meses de vida e, após tal período, a introdução de alimentos complementares, com manutenção do leite materno até os dois anos de idade ou mais, sendo ainda desincentivada a oferta de alimentos industrializados nos primeiros anos de vida.^2^

Apesar dos benefícios indiscutíveis da aplicação prática de tais recomendações, inúmeros estudos têm revelado que a sociedade contemporânea converge para padrões dietéticos inadequados, com impacto na introdução de alimentos industrializados e ultraprocessados precocemente na dieta infantil.^3^^,^ 4 ^,^ 5 ^and^ 6 Esta realidade é consequência direta da inserção da mulher no mercado de trabalho, associada à falta de tempo para o preparo de alimentos e à confiança depositada nos produtos veiculados pela mídia e associados por esta ao público infantil.^7^

Especificamente no tocante aos alimentos líquidos, observa-se aumento no consumo de bebidas artificias, como refrigerantes e sucos industrializados. Moodie *et al* 7 compilaram tendências na aquisição de refrigerantes em países de baixa e média renda, incluindo o Brasil, e países de alta renda, revelando enfático crescimento anual do volume per capita consumido entre os anos e 1997 e 2009, com aumento de 5,2% nos países de baixa e média renda e 2,4% nos de alta renda, demonstrando que se trata de problema global quee independe da situação socioeconômica e cultural.

Ressalta-se que, além dos prejuízos imediatos decorrente do consumo dessas bebidas como comprometimento da ingestão de leite materno e outros alimentos saudáveis e da adequação nutricional de micronutrientes, a sua manutenção na dieta habitual pode ter impacto em médio e longo prazo no incremento do sobrepeso, obesidade e doenças crônicas associadas,^8^ tal como verificado por Boynton *et al* 9 em 548 crianças de Massachussets, cujo Índice de Massa Corporal (IMC) e prevalência de obesidade aumentaram para cada porção adicional de bebidas que continha açúcar de adição.

Diante do exposto, o presente estudo teve como objetivo identificar a idade de introdução do refrigerante e de sucos industrializados na dieta de lactentes matriculados em berçários de creches públicas e comparar as composições nutricionais dessas bebidas com as do suco de fruta natural.

## Métodos

Trata-se de um estudo observacional múltiplo, com a primeira observação no segundo semestre do ano de 2007 e a segunda, no segundo semestre de 2010. O trabalho foi desenvolvido nos berçários de oito creches públicas pertencentes à Coordenadoria de Educação do bairro de Santo Amaro na Cidade de São Paulo, que fizeram parte do Projeto Crecheficiente - "Impacto do treinamento de educadoras de creches públicas/filantrópicas nas práticas higiênico-dietéticas e na saúde/nutrição dos lactentes", cujos objetivos foram capacitar, aperfeiçoar e atualizar os educadores de creches quanto aos cuidados de saúde e de nutrição oferecidos aos lactentes e avaliar a aquisição de conhecimentos pelos educadores relacionados às atividades por eles desenvolvidas. O processo de seleção das creches e os critérios adotados estão descritos em outra publicação.^10^

Das oito creches selecionadas para o estudo de 2007, uma foi excluída em 2010 por não apresentar interesse em participar da pesquisa no período de coleta dos dados. O estudo foi composto por todas as crianças matriculadas, totalizando 636 crianças, sendo 270 em 2007 e 366 em 2010, de ambos os sexos, com faixa etária entre 4 e 38 meses, que frequentavam regularmente os berçários das creches selecionadas e que foram autorizadas pelos pais ou responsáveis a participar da pesquisa, mediante assinatura do termo de consentimento informado, livre e esclarecido. Acrescenta-se que, visando uniformizar a análise dos dados, optou-se por analisar conjuntamente os dados das observações transversais de 2007 e de 2010, uma vez que não foi objetivo deste estudo a comparação dos dados coletados e sim a constituição de ampla amostra com todos os lactentes frequentadores dos berçários das creches estudadas.

A introdução dos dois alimentos, refrigerante e suco industrializado, foi avaliada a partir de informações coletadas por meio de questionário estruturado, pré-codificado e pré-testado quanto às suas validades de conteúdo e de construto. Registrou-se a idade em meses de introdução do refrigerante e do suco industrializado, não sendo investigadas as categorias dos sucos. A coleta de dados foi realizada, em 2007 e 2010, por quatro nutricionistas, devidamente treinadas que entrevistaram os pais ou responsáveis pelas crianças nas creches, em dias previamente agendados.

Visando uniformizar o preenchimento desse instrumento, elaborou-se um manual com orientações aos entrevistadores e codificação das variáveis. Analisaram-se os dados quanto à sua consistência interna, seguida da dupla digitação e validação. Realizaram-se as análises estatísticas por meio do programa Epi-Info 7, 2012 (Centers for Disease Control and Prevention, Georgia, United States). Para determinar a idade de introdução do refrigerante e suco industrializado utilizaram-se a frequência percentual acumulada nas faixas de idade de 0 a 6, 7 a 12 e 12 a 36 meses.

Avaliaram-se as composições centesimais do refrigerante e suco industrializado, comparando-as com as do suco natural, para valor energético, carboidrato total, fibra, vitamina C e sódio. Selecionou-se, para todas as bebidas, o sabor laranja, por tratar-se do mais consumido no Brasil.^11^ A composição centesimal do suco natural foi obtida, por meio de consulta à Tabela Brasileira de Composição de Alimentos.^12^ Para definir a composição centesimal dos alimentos industrializados, utilizaram-se as médias das informações nutricionais contidas nos rótulos de cinco marcas mais consumidas dos dois produtos,^13^ sendo que para a categoria do suco foram considerados os tipos: preparado sólido para refresco artificial, néctares e refrescos adoçados.^14^

O presente estudo foi aprovado pelo Comitê de Ética em Pesquisa da Universidade Federal de São Paulo (Unifesp) (CEP 0471/10).

## Resultados

A mediana da idade das crianças estudadas foi de 23 meses. Do total de crianças estudadas (n=636), observou-se predominância do sexo masculino (55,7%) e maior proporção de mães na faixa etária de 20 a 35 anos, com média de idade de 28±6,5 anos. Quanto à escolaridade materna, verificou-se que 36% das mães tinham menos de oito anos de estudo (tabela 1).

**Tabela 1:** Características demográficas e socioeconômicas de crianças estudadas

	n	%	Mediana
*Idade da criança*			
0 a 6 meses	4	0,6	23 meses
7 a 12 meses	50	7,9	
13 a 36 meses	577	91,3	
≥36 meses	1	0,2	

*Sexo*			
Masculino	354	55,7	-

*Idade da mãe*			
<20 anos	51	8	28 anos

*Escolaridade da mãe*			
<8 anos	171	26,9	10 anos

*Renda familiar (SM)*			
<1	21	3,3	
1.0-2.0	265	41,9	1,9 SM
2.0-3.0	137	21,6	
≥3.0	210	33,2	

SM, salário mínimo em reais vigente no período dos estudos, equivalente a 380 reais em 2007 e a 510 reais em 2010.

A tabela 2 apresenta a frequência acumulada (%) por idade da introdução do refrigerante e do suco industrializado, bem como a média e o desvio padrão da idade de introdução. Nota-se que, para mais da metade das crianças estudadas, ofereceu-se refrigerante e suco industrializado até o final do primeiro ano de vida, sendo que menos de 10% das crianças não haviam consumido essas bebidas até os 36 meses.

**Tabela 2:** Percentuais cumulativos por faixa etária, média e desvio-padrão da introdução de refrigerante e suco industrializado dos 636 lactentes avaliados

		Refrigerante	Sucos industrializados^b^
Meses na introdução			
0-6	%	7,4	14,3
7-12	%^a^	53,8	62,9
12-36	%^a^	90,4	91,8
Não introduziu	%	9,3	7,9
Não informado	%	0,3	0,3
Média±DP	meses	15,9±7,8	14,0±7,9

a Percentual acumulado.

b Referente às cinco marcas mais consumidas de preparado sólido para refresco, néctares e refrescos adoçados.

Na tabela 3, tem-se a composição nutricional do suco de laranja e, em média e desvio-padrão, do refrigerante e do suco industrializado. A comparação das composições nutricionais estão expressas na figura 1, onde constata-se que os refrigerantes e sucos artificiais contêm quantidades de 9-13 vezes superiores de sódio e 15 vezes inferiores de vitamina C, quando comparados ao suco natural. Sendo ainda inexistente a oferta de fibra em ambas bebidas artificiais.

**Tabela 3:** Valor energético, açúcar, fibra, vitamina C e sódio em média ± desvio padrão, na composição centesimal do suco de laranja natural, sucos industrializados e refrigerantes12, 13-14

	Suco natural^a^	Sucos industrializado^b^	Refrigerantes
Valor energético (kcal)	39	30,2±18,4	42,7±1,8
Açúcar (g)	8,6	7,5±5,3	10,8±0,6
Fibra (g)	0,4	0	0
Vitamina C (mg)	41,3	11,9±10,2	0
Sódio (mg)	Traço^c^	9,8±6,1	7,4±1,9

a Suco natural de laranja.

b Referente às cinco marcas mais consumidas de preparado sólido para refresco, néctares e refrescos adoçados.

c Traços, valores abaixo dos limites de quantificáveis.

**Figura 1:** Comparação de valor energético, açúcar, fibra, vitamina C e sódio, na composição centesimal do suco de fruta natural, sucos industrializados e refrigerantes.12, 13-14

## Discussão

Deve-se destacar que a decisão pela comparação do refrigerante e suco industrializado com o suco natural decorre do fato deste estar inserido entre os alimentos líquidos recomendados na alimentação complementar, ressaltado, porém que, cumprindo a recomendação do Ministério da Saúde para a prática do aleitamento materno, o suco também não deve ser oferecido antes dos seis meses de idade.^2^

Os resultados apresentados revelaram introdução precoce e inoportuna de refrigerantes e sucos artificiais na dieta das crianças estudadas, sendo que mais da metade os consumia antes do primeiro ano de vida. Foi ainda constatado que 7,4 e 14,3% das mães ofertaram respectivamente refrigerante e suco industrializado antes do sexto mês de vida, porcentagens significativas, uma vez que, neste período, recomenda-se a oferta exclusiva do leite materno. Se a introdução de alimentos complementares precocemente, independente de sua composição, já é referida na literatura como fator de risco para redução da duração e frequência do aleitamento materno,^2^ interação na absorção de nutrientes, risco para diarreias e doenças respiratórias, mortalidade infantil, e impacto no crescimento,^15^ quando a introdução refere-se a alimentos obesogênicos, os riscos à saúde são ainda mais graves, sobretudo no que concerne à predisposição para obesidade e doenças crônicas não transmissíveis (DCNT), incluindo diabetes, hipertensão arterial e doenças cardiovasculares.

Os dados supracitados refletem tendências de comportamento alimentar da sociedade contemporânea. Constata-se que, com a abertura da economia e a estabilização monetária, o mercado consumidor se ampliou.^16^ Simultaneamente ao aumento do poder aquisitivo, o preço real dos alimentos industrializados declinou, favorecendo a maior participação dos estratos sociais de menor renda. De maneira geral, à medida que aumenta a renda per capita de um país, aumenta o grau de sofisticação no consumo de alimentos, optando-se pelos mais elaborados, como os industrializados.^16^ ^and^ 17

Corroborando esta asserção, dados da Pesquisa de Orçamento Familar 2008-2009^18^ revelaram que o consumo de refrigerantes e sucos/refrescos/sucos em pó reconstituídos estiveram entre as maiores médias de consumo diário per capita, sendo de 94,7 e 145 mL/dia, respectivamente, sem diferenças entre as faixas de renda, associando-se ao menor consumo de vitamina C e de fibra e maior de energia e sódio. Por outro lado, a quantidade recomendada de frutas e hortaliças/dia não é atingida nem no percentil 90 da população brasileira, refletindo a preferência por industrializados. Especificamente no público infantil, Spinelli *et al* 5 verificaram a presença de alimentos considerados supérfluos na dieta de 400 crianças menores de um ano atendidas em Unidades Básicas de Saúde de um município da grande São Paulo e constataram que 58,9% das crianças já consumiam refrigerante, sendo que 14,5% o faziam entre os quatro e seis meses de idade.

De forma semelhante, em estudo prospectivo, Caetano *et al* 4 identificaram, por meio de registro alimentar de sete dias aplicado aos responsáveis, em amostras de lactentes residentes em Curitiba, São Paulo e Recife, frequência de ingestão semanal de refrigerante de 9% e de suco industrializado equivalente a 20,7%. Tais achados, em adição à presença de outros alimentos inapropriados para a idade, refletem inadequação quantitativa de micronutrientes superior a 15%, 40% e 10% para cálcio, ferro e vitamina C, respectivamente.

Ainda, Silva *et al*, 6 analisando dados provenientes de pesquisas de base populacional brasileira, constataram que, entre as 3789 crianças menores de cinco anos, 70% havia consumido refrigerante e suco industrializado pelo menos uma vez nos sete dias prévios à pesquisa, sendo o consumo diário referido por 22% da criancas estudadas. Os autores destacaram que a prevalência foi superior entre as residentes da zona urbana, justificada possivelmente pela recente comercialização de grande variedade des tais bebidas, maior facilidade de acesso, assim como pelo apelo das campanhas publicitárias.

Analisando os dados do presente estudo sob o enfoque da tenra idade de introdução, estes tornam-se ainda mais inquietantes, se considerado a tendência de aumento no consumo de refrigerantes e sucos artificiais conforme o avançar dos anos,^19^ sugerindo que estas crianças podem manter-se expostas a estas bebidas de forma usual e ascendente durante seu crescimento.

A exemplificar, Dubois *et al* 20 acompanharam uma coorte de mais de duas mil crianças, entre dois e cinco anos e verificaram que a proporção daquelas que consumiam refrigerantes uma vez na semana entre as refeições aumentavam conforme a idade (42% aos 2,5 anos, 47% aos 3,5 anos, 48% aos 4,5 anos), discutindo a possível associação desta tendência com a maior autonomia das crianças, inserção na escola, com exposição às cantinas escolares, em redes públicas e privadas, e, acrescenta-se aqui, a possível crença dos pais dos efeitos prejudiciais minimizados em crianças maiores. Em concordância, Lopes *et al* 21 também apontaram, entre crianças de São Paulo, relação direta entre o estado nutricional e consumo de refrigerantes, sendo que 83,2% que bebiam refrigerante todos os finais de semana apresentavam sobrepeso e 76,6% obesidade.

Os dados supracitados podem ser justificados pela composição nutricional inadequada dessa bebida, destacando que a quantidade de carboidrato presente em 100 mL, correspondente a 10,8 g, consiste exclusivamente ao açúcar de adição, valor que corresponde a mais de 80% da recomendação diária proposta pela OMS para crianças de um a três anos de idade (13,3 g/dia).^22^ Embora a quantidade de açúcar adicionada aos sucos artificiais seja desconhecida, já que sua declaração no rótulo não é obrigatória,^23^ supõe-se que também estejam presentes em quantidades excessivas.

A definição do termo açúcar de adição inclui além dos monos e dissacarídeos, alguns oligossacarídeos e não considera os açúcares naturalmente presentes em alimentos, como é o caso das frutas. Esses açúcares são aqueles acrescentados em preparações e alimentos processados com o objetivo de provocar paladar agradável, melhor viscosidade, textura, cor e durabilidade. Incluem-se neste termo açúcar refinado, mascavo, xarope de milho rico em frutose, xarope de glicose, frutose líquida, edulcorante a base de frutose, mel e melaço.^24^ A sua ingestão associa-se à redução geral da qualidade da dieta, ocorrência precoce de sobrepeso e obesidade, desenvolvimento de doenças crônicas e seus fatores de risco^2^ bem como contribui para ocorrência de cárie dentária, fato comprovado por Biral *et al*, 25 que avaliaram as crianças inseridas na coleta de dados de 2007 do presente estudo e identificaram 77% delas com alguma alteração para o índice de cárie modificado (ceo-mod≥1) e 72,37% com placa bacteriana.

Assim, respaldada no papel deletério da elevada ingestão de açúcar e bebidas artificiais e tendo como foco particular a prevenção e controle do sobrepeso e cárie dental, a OMS propôs, em 2014, redução no limite máximo recomendado de açúcar, de 10 para 5% do consumo energético total diário.^21^ Contrariando tais perspectivas, a população brasileira, maior consumidora mundial de açúcar, possui ingestão estimada equivalente a 16,4% das calorias totais.^26^ Segundo Levyl *et al*, 26 a participação do açúcar de mesa nos últimos 15 anos foi reduzida, enquanto a contribuição do açúcar adicionado aos alimentos dobrou, especialmente por meio do consumo de refrigerantes e biscoitos.

Embora não seja de amplo conhecimento do consumidor mundial, a diferença entre suco, néctar e refresco está relacionada ao teor do suco de fruta presente na bebida envasada. Na categoria de néctar, a bebida envasada possui menor conteúdo de suco puro, pode conter adoçantes, corantes e conservantes, aditivos^14^ que geralmente são mais baratos do que os sólidos solúveis das frutas, razão pela qual tornam esta categoria mais acessível a uma faixa de consumo de renda per capita intermediária.^27^ A Instrução normativa do Ministério da Agricultura, Pecuária e Abastecimento (Mapa) publicada no Diário Oficial da União, em 2012, elevou de 30% para 50% o conteúdo mínimo de suco de laranja nas bebidas vendidas como néctar da fruta.^28^

Já na categoria de refresco, o conteúdo de suco na bebida envasada é de 30% em volume do suco natural. Nessas bebidas encontra-se uma quantidade maior de aditivos, tornando-as um produto de menor valor agregado, representando a porta de entrada para o consumo de bebidas de frutas industrializadas da população de menor renda.^27^ Os refrigerantes de laranja devem conter, obrigatoriamente, no mínimo 10% em volume do respectivo suco na sua concentração natural e os preparados sólidos para refresco artificial não possuem matéria prima de origem vegetal em sua composição.^14^ O desconhecimento destas definições e a falta de clareza dos rótulos destes alimentos podem confundir o consumidor que adquire tais bebidas sob a perspectiva ilusória de estar substituindo de forma adequada o suco natural ou até mesmo a fruta in natura.

No que se refere às quantidades de micronutrientes observadas para refrigerantes e sucos artificiais aqui analisados, aponta-se que a quantidade de vitamina C foi aproximadamente quatro vezes superior na composição centesimal do suco natural (41,3 mg), quando comparada ao suco artificial (11,9 mg), sendo nula a quantidade da vitamina no refrigerante, destacando-se que esta exerce papel importante na absorção do ferro não heme e no sistema imunológico. Ambas as bebidas não ofertam fibra alimentar.

Somado a esta questão, ressalta-se o potencial papel inibidor destas bebidas na absorção de outros micronutrientes. Os refrigerantes contêm em sua composição polifenóis, que também desempenham papel inibidor na absorção de ferro não heme,^29^ contribuindo, para o risco do desenvolvimento de anemia ferropriva, carência nutricional de importância mundial em Saúde Pública, sobretudo na faixa de idade das crianças do presente estudo. Especificamente na população aqui estudada, Konstantyner *et al* 10 encontraram prevalência de anemia de 51,9% (IC95%: 44,9-58,8%) em 2007.

O cálcio é outro micronutriente que pode sofrer prejuízos em decorrência da ingestão dessas bebidas. Há diversas razões para a hipótese que refrigerantes carbonados, particularmente de cola, podem associar-se com menor Densidade Mineral Óssea (DMO), ressaltando a presença da cafeína, identificada como fator de risco para osteoporose.^30^ O ácido fosfórico interfere na absorção do micronutriente e contribui para desequilíbrios que aumentam a sua excreção, além do xarope de milho, rico em frutose, usado para adoçar estas bebidas, possuem efeito ósseo negativo.^30^

Comprovando tais hipóteses, Tycker *et al*, 31 utilizando dados do Estudo de Osteoporose de Framinghan, examinaram associação entre consumo de bebidas carbonadas e densidade mineral óssea, com subdivisão entre os tipos de cola e não cola. Tais dados foram obtidos por meio de absorciometria de raio-X de dupla energia e aplicação de Questionário de Frequência de Consumo Alimentar. A Densidade Mineral Óssea (DMO) média de mulheres com ingestão diária de cola foi 3,7% menor no colo do fêmur e 5,4% no triângulo de Ward, quando comparadas às que consumiram <1 porção de cola/mês, com significância estatística (*p<*0.001-0.05).

Evidenciando ainda aspectos desfavoráveis do consumo precoce dessas bebidas, embora não esteja entre os objetivos do presente estudo, cumpre enfatizar que as bebidas artificiais possuem em sua composição aditivos alimentares que, segundo recomendações da OMS e Organização das Nações Unidas para Agricultura e Alimentação (FAO), não devem ser utilizados internacionalmente em produtos destinados a crianças menores de um ano.^32^ Nogueira^8^ avaliou o consumo de corantes por pré-escolares de creches públicas e privadas do Rio de Janeiro e constatou contribuição excessiva proveniente de refrigerantes, sucos de garrafa e refrescos, cuja ingestão foi maior entre aqueles de creches públicas, decorrente do baixo custo destes produtos.

Em adição aos malefícios associados ao aspecto nutricional dos sucos artificiais e refrigerantes, deve-se ainda enfatizar o fato dessas crianças estarem em processo de constituição dos hábitos alimentares. Embora a apetência pelo sabor doce seja inata, a sua consolidação é influenciada também pela experiência, ou seja, pelo consumo repetido de alimentos doces durante a primeira infância.^33^

Considerando, portanto, todos os efeitos prejudiciais supracitados, deve-se tentar compreender os fatores que contribuem para a oferta precoce de bebidas artificiais no contexto domiciliar. Além de, serem reflexo dos hábitos alimentares inadequados generalizado entre a população, são marcantes as modificações no perfil de aquisição e alimentos pelas famílias e no consumo de refeições pré-preparadas, reforçando ainda a influência da publicidade e a confiança depositada em produtos apresentados na mídia e anúncios publicitários, sobretudo decorrente da desinformação sobre os riscos para a saúde e nutrição associados ao consumo precoce e continuado destes alimentos.^7^

No tocante ao papel da publicidade de alimentos no incentivo ao consumo desses produtos, apesar da *American Dietetic Association* recomendar que crianças menores de dois anos não sejam expostas à televisão ou qualquer outra tela ou aparelhos eletrônicos, em média, elas assistem a programas televisionados por uma a duas horas por dia e 14% das crianças entre seis e 23 meses assistem duas ou mais horas por dia, 34 refletindo que essas crianças podem prosseguir sob o mesmo contexto da vulnerabilidade aos apelos publicitários dos alimentos veiculados pela mídia.

Diante desse cenário, foi publicado em 2012 o "Plano de ações estratégicas para enfrentamento das DCNT (2011-2022)", estabelecendo como principais ações a promoção da alimentação saudável, estando entre as metas para o seu alcance, o estabelecimento de acordos com a indústria para redução do sal e do açúcar dos alimentos por ela processados, além de restrições sobre o marketing de alimentos e bebidas com muito sal, gorduras e açúcar, especialmente para crianças.^35^ Ainda que o acordo entre o setor público e privado pareça uma estratégia promissora, Moodie *et al*, 7 ressaltam que a indústria vem traçando uma série de artifícios contrários aos programas e políticas de saúde pública, tal como o financiamento de pesquisas e congressos de saúde, patrocínio a eventos relacionados à prática de atividade física, opondo-se às regulamentações sob o argumento de que intervenções governamentais são coercivas e oprimem a liberdade e responsabilidade individual de escolha. Sob este enfoque e, consequentemente, com menor apoio da sociedade civil, a adoção de medidas regulatórias, como advertência de restrição, aumento das taxas e impostos sob produtos industrializados, torna-se mais cautelosa e até mesmo inviabilizada.

No que concerne às limitações do presente estudo, por tratar-se de um delineamento transversal, os dados relacionados à introdução decorrem de informações fornecidas pelas mães ou responsáveis de maneira recordatória, o que poderia conceber imprecisões ou viés. Porém, acredita-se que os resultados não foram substancialmente influenciados, uma vez que a introdução ocorreu alguns meses antes da entrevista. Outra limitação inclui a utilização de dados provenientes de Tabelas de Composição e Rótulos Nutricionais dos alimentos, ressaltando que a Agência Nacional de Vigilância Sanitária (ANVISA) permite uma variação do teor de nutriente, entre a tabela nutricional dos rótulos e o valor real, de mais ou menos 20%.^22^

Por fim, pode-se concluir que a introdução de refrigerantes e sucos industrializados na dieta dos lactentes é inoportuna e precoce, bem como, quando comparados ao suco de fruta natural, possuem composição nutricional inferior. Tais achados sugerem a necessidade de medidas, fundamentadas em estratégias de educação alimentar e nutricional voltadas aos pais, crianças e demais funcionários atuantes no contexto das creches, que contribuam para reduzir o consumo de bebidas artificiais como forma de promover a formação e a manutenção de hábitos alimentares saudáveis, contribuindo positivamente para o crescimento e o desenvolvimento adequados das crianças e prevenindo em curto, médio e longo prazo o incremento do sobrepeso, da obesidade e das doenças crônicas.
